# Association of State Medicaid Expansion Status With Rates of Suicide Among US Adults

**DOI:** 10.1001/jamanetworkopen.2022.17228

**Published:** 2022-06-15

**Authors:** Hetal Patel, Justin Barnes, Nosayaba Osazuwa-Peters, Laura Jean Bierut

**Affiliations:** 1 Department of Psychiatry, Washington University School of Medicine in St Louis, St Louis, Missouri; 2 Department of Radiation Oncology, Washington University School of Medicine in St Louis, St Louis, Missouri; 3 Deputy Editor, Diversity, Equity, and Inclusion, *JAMA Otolaryngology–Head and Neck Surgery*; 4 Department of Population Health Sciences, Duke University School of Medicine, Durham, North Carolina; 5 Department of Head and Neck Surgery & Communication Sciences, Duke University School of Medicine, Durham, North Carolina

## Abstract

**Importance:**

In the US, suicide is the 10th leading cause of death and a serious mental health emergency. National programs that address suicide list access to mental health care as key in prevention, and more large-scale policies are needed to improve access to mental health care and address this crisis. The Patient Protection and Affordable Care Act (ACA) Medicaid Expansion Program was implemented in several states with the goal of increasing access to the health care system.

**Objective:**

To compare changes in suicide rates in states that expanded Medicaid under the ACA vs states that did not.

**Design, Setting, and Participants:**

In this cross-sectional study, state-level mortality rates were obtained from the National Center for Health Statistics for US individuals aged 20 to 64 years from January 1, 2000, to December 31, 2018. Data analysis was performed from April 18, 2021, to April 15, 2022.

**Exposures:**

Changes in suicide mortality rates among nonelderly adults before and after Medicaid expansion in expansion and nonexpansion states were compared using adjusted difference-in-differences analyses via hierarchical bayesian linear regression.

**Main Outcomes and Measures:**

Suicide rates using death by suicide as the primary measure.

**Results:**

Of the total population at risk for suicide, 50.4% were female, 13.3% were Black, 79.5% were White, and 7.2% were of other races. The analytic data set contained suicide mortality data for 2907 state-age-year units covering the general US population. A total of 553 912 deaths by suicide occurred during the study period, with most occurring in White (496 219 [89.6%]) and male (429 580 [77.6%]) individuals. There were smaller increases in the suicide rate after 2014 in Medicaid expansion (2.56 per 100 000 increase) compared with nonexpansion states (3.10 per 100 000 increase). In adjusted difference-in-differences analysis, a significant decrease of −0.40 (95% credible interval, −0.66 to −0.14) suicides per 100 000 individuals was found, translating to 1818 suicides that were averted in 2015 to 2018.

**Conclusions and Relevance:**

In this cross-sectional study, although suicide rates increased in both groups, blunting of these rates occurred among nonelderly adults in the Medicaid expansion states compared with nonexpansion states. Because this difference may be linked to increased access to mental health care, policy makers should consider suicide prevention as a benefit of expanding access to health care.

## Key Points

**Question:**

What is the association of Medicaid expansion under the Patient Protection and Affordable Care Act with suicide rates?

**Findings:**

Although suicide rates increased from 2000 to 2018 across all states in this cross-sectional study, a statistically significant attenuation of −0.40 suicides per 100 000 people was found in Medicaid expansion states compared with nonexpansion states, translating to 1818 fewer suicides from 2015 to 2018.

**Meaning:**

These findings suggest that Medicaid expansion, which increases mental health coverage, is associated with a decrease in suicide mortality.

## Teaser

This cross-sectional study of state-level mortality rates from 2000 to 2018 assesses whether Medicaid expansion is associated with suicide rates after enactment of the Patient Protection and Affordable Care Act.

## Introduction

Suicide is the 10th leading cause of death in the US and the second leading cause of death in people aged 10 to 34 years (only secondary to unintentional injury), making suicide a national emergency.^1^ Suicides have been steadily increasing since 1999, with a mean increase of 1% per year from 1999 to 2006 and 2% per year from 2006 to 2018.^2^ The prevalence of suicide increased from 12.3 cases per 100 000 individuals in 2011 to 14.2 cases per 100 000 individuals in 2018.^3^ Deaths from suicide have resulted in a yearly expenditure of greater than $4 billion in combined medical costs and work loss costs.^4^

Suicidality is often a symptom of mental illnesses, and increasing treatment of mental illnesses and improving mental health care access is a target for suicide prevention.^5,6,7^ To reduce suicide incidence, the Centers for Disease Control and Prevention has recommended improving access to suicide-related health care and strengthening economic supports.^8^ One of the most important barriers to mental health care is lack of insurance coverage and financial resources, particularly for low-income patients.^8,9,10^ Under the Patient Protection and Affordable Care Act (ACA), states had the opportunity to broaden health care access to low-income adults. In 2014, a total of 26 states expanded Medicaid eligibility to all individuals at and 138% below the federal poverty level (expanding care to a key demographic characteristic: childless, nonelderly, nondisabled adults).^10^ By 2021, a total of 39 states had adopted Medicaid expansion.

Since expansion, among patients with mental illness and substance use disorder, lack of health care insurance decreased by 6.8% from 2014 to 2015 alone, and Medicaid enrollment increased by almost 5%.^11^ Mental health treatment significantly increased with the ACA expansion.^12^ Medicaid expansion has also been associated with positive self-reported general health, which may have been driven by positive changes in mental health, including decreases in total days in poor mental health, days limited by poor health, and depression.^12,13^ Several articles have highlighted benefits of Medicaid expansion, including increased insurance coverage, increased health care access, and decreased financial strain.^10,14^ Data have shown increased reporting of a personal physician, increased preventive visits, fewer medication lapses due to cost, and decreased likelihood of emergency department visits as a result of increased health care coverage.^15^

Previous Medicaid expansions have shown an association with improvement in all-cause mortality, cardiovascular-related death, and breast, lung, and colorectal cancer mortality.^16^ Similarly, early data have also shown associations between Medicaid expansions under the ACA and decreased mortality overall and from cardiovascular, cancer, and other causes.^17,18,19,20,21,22^ Despite significant findings of the mortality-related benefits of Medicaid expansion, data related to the effect on mental health–related mortality remain limited. A recent study^23^ that included 8 Medicaid expansion states compared with 7 nonexpansion states demonstrated a modest trend between expansion and improved suicide mortality (1.2 fewer suicides per 100 000 individuals; 95% CI, −2.5 to 0.1), although those findings were not statistically significant. Our report builds on the aforementioned study^23^ by using data from all 50 states and Washington, DC. Our objective was to determine whether Medicaid expansion is associated with change in suicide mortality in the US.

## Methods

For this cross-sectional study, mortality data for patients who died from suicide at 20 to 64 years of age from January 1, 2000, to December 31, 2018, were collected from the National Center for Health Statistics, which is based on *International Statistical Classification of Diseases and Related Health Problems, Tenth Revision* codes from death certificates. These population-level data reflect all documented deaths from suicide in the US and represent the number of suicides (per 100 000 individuals) within a defined population of individuals aged 20 to 64 years (see the eAppendix in the Supplement for additional information regarding the data structure). We collected age-adjusted suicide mortality information for each combination of state, year, and age group (20-29, 30-44, and 45-64 years)^23^ for our primary analyses. Note that age information was restricted to 5-year groupings defined by the National Center for Health Statistics. For subgroup analyses, we also obtained information by race (White, Black, or other) and sex. Additional data on race and ethnicity were not available. More complete disaggregation of the data (ie, by year, state, age, race, and sex) for the primary analyses was not performed because of the censoring of data when the number of suicides for a given population was less than 10. Data were obtained using SEER*Stat software, version 8.3.9 (National Cancer Institute). Because of the aggregate nature of these publicly available data, the study was determined to be non–human subjects research by the Washington University in St Louis Institutional Review Board. This study followed the Strengthening the Reporting of Observational Studies in Epidemiology (STROBE) reporting guideline.

### Statistical Analysis

Analyses were conducted between April 18, 2021, and April 15, 2022. We used a difference-in-differences (DID) analysis to compare changes in suicide from before to after Medicaid expansion in expansion vs nonexpansion states based on Medicaid expansion status at the end of the study period (December 31, 2018). Because most expansion states expanded during 2014, we defined the postexpansion period as 2015 to 2018. However, we defined the start of the postexpansion period for states that expanded Medicaid from 2015 to 2018 (Alaska, Indiana, Louisiana, Montana, and Pennsylvania) as the year after the date of expansion enactment.

We implemented the DID analyses with hierarchical bayesian linear regression models accounting for year (continuous), age group, state effects, and time-varying state-level factors, including the percentage of non-Hispanic White individuals, the percentage of individuals living in poverty, the percentage of individuals without a high school education, the percentage of unemployed individuals, the number of opioid prescribing laws, and the number of firearm laws.^24^ The covariates were determined a priori because of their suspected associations with suicide rates and/or socioeconomic status and Medicaid eligibility. Because of the state-level structure of the data with repeated measures over time, the model accounted for serial autocorrelation and allowed for state-specific temporal trends.^25^ The marginal posterior distribution was used to obtain the DID estimate (mean), 95% credible interval (CrI; 2.5th and 97.5th percentiles), and the 1-tailed probability of the estimate being greater than 0 (Pr[Est >0]). Because this probability is 1-sided, a Pr(Est >0)<.025 was required for statistical significance. See the eMethods and eTable 1 in the Supplement for more detailed information about the modeling approach.

The plausibility of the parallel trends assumption was assessed by an event study plot, which compares the difference in suicide rates between expansion and nonexpansion states over time (eFigure 1 in the Supplement). We also conducted sensitivity analyses with the inclusion of a group-specific linear trend (eTable 2 and eFigure 2 in the Supplement).^25^ We conducted analyses overall as well as by age, sex, and race subgroups to determine whether Medicaid expansion effects were more prominent among a particular subgroup of the population. Data analysis was performed using R software, version 4.1.0 (R Foundation for Statistical Computing) and the R2jags and superdiag packages.

## Results

Of the total population at risk for suicide, 50.4% were female, 49.6% were male, 13.3% were Black, 79.5% were White, and 7.2% were of other races (American Indian or Alaska Native, Asian, and Native Hawaiian or Other Pacific Islander). Our analytic data set contained suicide mortality data for 2907 state-age-year units (51 states [including Washington, DC] × 3 age groups ×19 years of data). During the study period from 2000 to 2018, a total of 553 912 deaths by suicide among those 20 to 64 years of age occurred in the US, with most suicides occurring in White (496 219 [89.6%]) and male (429 580 [77.6%]) individuals (Table 1). The population at risk was the general US population aged 20 to 64 years. The population and suicide mortality distributions by age and sex were similar in Medicaid expansion and nonexpansion states, but Medicaid expansion states had lower percentages of Black individuals and higher percentages of other races compared with nonexpansion states. Unemployment and educational levels were similar between the state groups. However, poverty rates and the percentage of non-Hispanic White individuals were slightly higher in nonexpansion states. Opioid prescribing laws and firearm control laws increased more in the Medicaid expansion states during the study period.

**Table 1.** Characteristics of the Study Population

Characteristic	Total	Medicaid expansion states^a^		Nonexpansion states^b^	
		2000-2013^c^	2015-2018	2011-2013^c^	2015-2018
Population at risk, %					
Overall	100	100	100	100	100
Age, y					
20-29	23.2	22.9	23.6	23.4	23.7
30-44	34.4	35.1	32.4	35.1	32.9
45-64	42.4	42.0	44.0	41.5	43.4
Sex					
Male	49.6	49.7	49.8	49.5	49.6
Female	50.4	50.3	50.2	50.5	50.4
Race					
Black	13.3	11.1	11.9	16.4	17.7
White	79.5	80.7	77.9	79.5	76.9
Other^d^	7.2	8.2	10.2	4.2	5.4
Suicide mortality events, No. (%)					
Overall	553 912 (100)	226 271 (100)	81 350 (100)	155 006 (100)	58 471 (100)
Age, y					
20-29	111 775 (20.2)	44 624 (19.7)	17 395 (21.4)	30 597 (19.7)	12 631 (21.6)
30-44	184 597 (33.3)	77 204 (34.1)	25 128 (30.9)	53 720 (34.7)	18 553 (31.7)
45-64	257 540 (46.5)	104 443 (46.2)	38 827 (47.7)	70 689 (45.6)	27 287 (46.7)
Sex					
Male	429 580 (77.6)	177 356 (78.4)	62 340 (76.6)	120 101 (77.5)	44 742 (76.5)
Female	124 332 (22.4)	48 915 (21.6)	19 010 (23.4)	34 905 (22.5)	13 729 (23.5)
Race					
Black	35 779 (6.5)	13 022 (5.8)	4998 (6.1)	11 148 (7.2)	4589 (7.8)
White	496 219 (89.6)	202 507 (89.5)	71673 (88.1)	140 526 (90.7)	52 069 (89.1)
Other^d^	21 914 (4.0)	10 742 (4.7)	4679 (5.8)	3332 (2.1)	1813 (3.1)
State characteristics					
State-age-year group observations, No.	2907	1461	363	855	228
Unemployed, %	5.5	5.9	4.6	5.4	4.3
Less than high school education, %	14.4	15.0	10.9	15.8	11.5
Poverty, %	13.3	13.0	12.5	14.0	13.3
Non-Hispanic White, %	69.5	69.6	63.7	72.5	67.4
Opioid prescribing laws, mean No.	3.2	2.5	4.2	3.4	4.7
Firearm laws, mean No.	20.1	21.0	37.5	13.0	12.9

^a^ Medicaid expansion states included Arizona, Arkansas, California, Colorado, Connecticut, Delaware, Hawaii, Illinois, Iowa, Kentucky, Maryland, Massachusetts, Michigan, Minnesota, Nevada, New Hampshire, New Jersey, New Mexico, New York, North Dakota, Ohio, Oregon, Rhode Island, Vermont, Washington, Washington, DC, and West Virginia, which all expanded Medicaid during 2014. Note that Alaska, Indiana, Louisiana, Montana, and Pennsylvania are also Medicaid expansion states, although these states enacted expansions later (2015-2018).

^b^ Nonexpansion states are those that have not expanded Medicaid or expanded after 2018, including Alabama, Florida, Georgia, Idaho, Kansas, Maine, Mississippi, Missouri, Nebraska, North Carolina, Oklahoma, South Carolina, South Dakota, Tennessee, Texas, Utah, Virginia, and Wyoming.

^c^ Observations from 2014, the year during which most Medicaid expansion states enacted expansion, were included in our final adjusted analyses and are given in the total column.

^d^ Other subgroup includes data for the following racial groups: American Indian or Alaska Native, Asian, and Native Hawaiian or Other Pacific Islander.

Medicaid expansion states had a lower incidence of suicide (13.94 per 100 000 individuals) compared with the nonexpansion states (16.67 per 100 000 individuals) in the preexpansion period (2011-2013). Suicide rates increased similarly in Medicaid expansion and nonexpansion states from 2000 through 2013 (Figure; see also eFigure 1 in the Supplement for the event study plot), suggesting that the assumption of parallel trends is plausible. However, an attenuation of the increase occurred after 2014 in Medicaid expansion states (2.56 per 100 000 increase) compared with nonexpansion states (3.10 per 100 000 increase) (Figure and Table 2). In adjusted DID analyses, a significant decrease of −0.40 (95% CrI, −0.66 to −0.14; Pr[Est >0]<.001) suicides per 100 000 individuals occurred in Medicaid expansion compared with nonexpansion states, which translates to 1818 suicides that were averted in Medicaid expansion states after the expansions.

**Figure.**
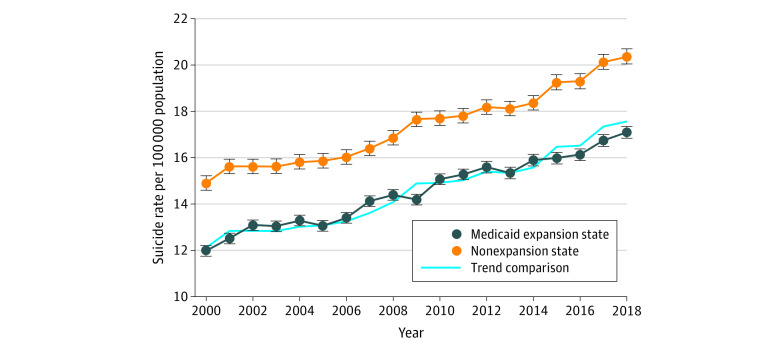
Temporal Trends in Suicide Rates by State Medicaid Expansion Status Suicide rates increased generally during the study period, with similar increases in Medicaid expansion and nonexpansion states from 2000 to 2013. However, after 2014, the increases were smaller in Medicaid expansion compared with nonexpansion states. The trend comparison was included for visual reference and is equal to the suicide rate trends of the nonexpansion states translated down such that the trend comparison in 2013 was equal to the suicide rate in Medicaid expansion states the same year. Note that our adjusted difference-in-differences analyses include states that expanded Medicaid from 2015 to 2018 (Alaska, Indiana, Louisiana, Montana, and Pennsylvania), whereas these states were excluded from this visualization of the data. Error bars indicate 95% credible intervals.

**Table 2.** Changes in Suicide Rates in Medicaid Expansion and Nonexpansion States

Characteristics	Suicide mortality rate per 100 000 individuals							Difference-in differences analyses		
	All states, 2000-2018	Expansion states^a^			Nonexpansion states^b^			Unadjusted estimates	Adjusted estimates^c^	
		2000-2013^d^	2015-2018	Change	2000-2013^d^	2015-2018	Change		Estimate (95% CrI)	Pr(Est >0)
Total	16.82	13.94	16.49	2.56	16.67	19.77	3.10	−0.54	−0.40 (−0.66 to −0.14)	<.001
Age, y										
20-29	14.72	11.92	14.95	3.03	14.09	18.10	4.02	−0.99	−0.52 (−1.00 to −0.05)	.02
30-44	16.18	13.48	15.77	2.29	16.46	19.15	2.68	−0.40	−0.30 (−0.72 to 0.09)	.07
45-64	18.74	15.60	18.16	2.56	18.41	21.40	2.99	−0.43	−0.04 (−0.40 to 0.31)	.42
Sex										
Male	26.03	21.95	25.3	3.35	26.06	30.49	4.43	−1.07	−0.41 (−0.86 to 0.03)	.04
Female	7.71	6.02	7.70	1.68	7.44	9.19	1.75	−0.07	−0.17 (−0.41 to 0.06)	.29
Race										
Black	7.90	7.25	8.61	1.37	7.21	8.69	1.48	−0.11	0.18 (−0.17 to 0.54)	.84
White	19.09	15.46	18.66	3.20	18.98	22.86	3.88	−0.68	−0.39 (−0.71 to −0.09)	.005
Other^e^	9.04	7.81	9.17	1.35	8.52	11.18	2.66	−1.30	−0.37 (−1.05 to 0.21)	.13

Abbreviations: CrI, credible interval; Pr(Est >0), 1-tailed probability of the estimate being greater than 0 (Pr[Est >0]<.025 indicates statistical significance).

^a^ Medicaid expansion states included Arizona, Arkansas, California, Colorado, Connecticut, Delaware, Hawaii, Illinois, Iowa, Kentucky, Maryland, Massachusetts, Michigan, Minnesota, Nevada, New Hampshire, New Jersey, New Mexico, New York, North Dakota, Ohio, Oregon, Rhode Island, Vermont, Washington, Washington, DC, and West Virginia. Note that Alaska, Indiana, Louisiana, Montana, and Pennsylvania expanded in 2015 to 2018 and were included as Medicaid expansion states in our adjusted difference-in-differences analyses (with staggered postexpansion period definitions), although they were excluded from the suicide mortality rate information and unadjusted analyses because of differing postexpansion period definitions.

^b^ Nonexpansion states are those that have not expanded Medicaid or expanded after 2018, including Alabama, Florida, Georgia, Idaho, Kansas, Maine, Mississippi, Missouri, Nebraska, North Carolina, Oklahoma, South Carolina, South Dakota, Tennessee, Texas, Utah, Virginia, and Wyoming.

^c^ Adjusted estimates are based on a hierarchical bayesian linear regression model accounting for covariates including state fixed effects and state-specific trends, age, and state-level unemployment, poverty, educational level, opioid prescribing laws, and firearm laws. The model also accounts for correlation between repeated measures over time and age groups within state clusters.

^d^ Observations from 2014, the year during which most Medicaid expansion states enacted expansion, were included in our final adjusted models and in the summary statistics for 2000 to 2018.

^e^ Other subgroup includes data for the following racial groups: American Indian or Alaska Native, Asian, and Native Hawaiian or Other Pacific Islander.

In subgroup analyses, the largest qualitative expansion-associated mitigation in suicide rates occurred among the population 20 to 29 years of age (−0.52 suicides per 100 000; 95% CrI, −1.00 to −0.05; Pr[Est >0] = .02) (Table 2). Notably, minimal expansion-associated changes were found among those aged 45 to 64 years (−0.04 suicides per 100 000 individuals; 95% CrI, −0.40 to 0.31; Pr[Est >0] = 0.42), women (−0.17 suicides per 100 000 individuals; 95% CrI, −0.41 to 0.06; Pr[Est >0] = 0.29), and Black individuals (0.18 suicides per 100 000 individuals; 95% CrI, −0.17 to 0.54; Pr[Est >0] = 0.84). In sensitivity analyses using group-specific trends, the DID results were similar, and the group-specific trend effects were small and statistically nonsignificant for all analyses except the subgroup analysis of the White population (eTable 2 in the Supplement).

## Discussion

We aimed to describe population-level differences in suicide mortality rate among nonelderly individuals in the US based on Medicaid expansion. We found a blunting in suicide mortality among people aged 20 to 64 years in Medicaid expansion states compared with nonexpansion states. Suicide has increased during the past 20 years, and although suicide continued to increase in both expansion and nonexpansion groups, there was a statistically significant improvement of −0.40 suicides per 100 000 individuals associated with the Medicaid expansion states. In other words, although both groups continue to have increased suicide rates per year, the states that adopted Medicaid expansion had an attenuation in the suicide increase compared with the nonexpansion states. The largest expansion-associated attenuation in suicide mortality was among those aged 20 to 29 years, and expansion-associated changes for those aged 45 to 64 years, women, and Black individuals were notably minimal. To our knowledge, this is the first report to describe significant changes in suicide mortality associated with Medicaid expansion using comprehensive data from all 50 states and Washington, DC.

The trend of increasing suicides in the past 2 decades has been part of a larger phenomenon coined by 2 Princeton economists, Anne Case and Angus Deaton, as “deaths of despair.”^26,27^ The economists theorized that this shift was a result of diminishing jobs, unstable families, and systemic and structural inequalities in education, income, and housing.^26,27,28^ An important facet detailed in their work is the indirect financial burden of the current US health care system on individuals and families fueling despair in the US and, as a result, deaths.^26^ With the increase of private health insurance coverage, mental health and substance use–related benefits became more limited compared with other services, including caps on the number of covered inpatient days for mental illness and outpatient visits as well as higher copayments for mental health services than for other services.^29^ The ACA aimed to improve access to mental health services by expanding requirements for employer-sponsored health plans and Medicaid-managed care plans to have mental health coverage comparable to medical coverage in terms of financial requirements and treatment limits.^30,31,32,33^ Approximately 30% of individuals who gained coverage through Medicaid expansion had a mental or substance use disorder.^32^ The ACA expansion was effective in improving access to mental health care, which increased for an estimated 62.5 million individuals.^13,31^ Low-income and middle-income families had greater reductions in health care–related financial burden with expansion.^34,35^ Thus, the finding that Medicaid expansion, which can reduce financial strain related to illness and provide mental health coverage,^13,31^ is associated with a reduction in suicide is consistent with this hypothesis of deaths of despair.

Although suicides have increased in the past 20 years across the US, the risk of suicide varies by state, and many factors contribute to differences in the incidence of suicide. Major suicide risk factors include mental illness, substance use, relationship trouble and poor social support, poor physical health, access to firearms, financial stressors, and legal stressors.^5,6,7,36^ The prevalence of these factors is affected by community- and state-level demographic characteristics, resource distribution, and political influence. For example, states with stricter gun laws and the lowest prevalence of gun ownership also tend to rank among the states with the lowest suicide incidence.^36^ Conversely, the top 3 states for gun ownership are among the states with the highest suicide rates.^36^ Similar to gun control laws, other policies, such as opioid prescribing laws, also vary state to state and may in part account for the baseline differences in suicide rates among states.^37^ Although we account for the number of opioid prescribing laws and firearm control laws by state in our analysis, we acknowledge that this consideration is simply a surrogate measure of opioid and gun control.

A similar study by Austin et al^23^ analyzed 2005 to 2017 suicide data from the National Violent Death Reporting System, with results suggesting improvement in suicide rates associated with Medicaid expansion. Data were compiled from 8 expansion states and 7 nonexpansion states, revealing that changes in suicide rates in nonelderly adults in Medicaid expansion states differed by −1.2 per 100 000 individuals compared with nonexpansion states, although that estimate was nonsignificant with a relatively wide confidence interval (−2.5 to +0.1). In contrast to the findings of Austin et al,^23^ our analyses of the total US population yielded a statistically significant association between Medicaid expansion and suicide mortality, even though the magnitude of association was more modest. In that study, subgroup analyses revealed significant changes in suicide deaths among both sexes, those aged 30 to 44 years, non-Hispanic White individuals, and individuals without a college degree. No change occurred in suicide rates among individuals aged 18 to 29 or 45 to 64 years, among individuals of races other than White, or among Hispanic individuals.^23^ Similarly, we found nonsignificant expansion-associated changes in suicide rates among Black individuals and individuals aged 45 to 64 years. However, in contrast to the results of the study by Austin et al,^23^ our data support significant expansion-associated attenuations in the suicide rate among the total US population and among those aged 20 to 29 years, which was the largest of all the subgroups tested. The reasons for the differing results and conclusions are unclear but may be related to our inability to account for ethnicity as well as differing study populations (all 50 states in the current study in contrast to a small subset in the study by Austin et al).^23^ Of note, however, a recent report^38^ of the associations of Medicaid expansion and the risk of suicide among patients with newly diagnosed cancer also found that expansion-associated attenuation in suicide risk was largely limited to young adults.

In our subgroup analyses by race, only the White group had associated attenuations in suicide rates after ACA expansion. Of interest, prior data show racial and ethnic minority groups had higher gains in private and public coverage and decreasing coverage disparities subsequent to ACA implementation.^39,40,41^ However, improvements in health care outcomes observed with Medicaid expansion have not necessarily translated to improved health status for racial and ethnic minority groups. Black individuals continue to report the highest rates of poor to fair health status, and a significant change has not occurred in the gap between reported health status of Black and Hispanic individuals compared with White individuals since 1999 despite broader insurance coverage.^42^ Similarly, the gap in the rates of psychological distress between non-White individuals compared with White individuals did not significantly change from 1999 to 2018.^42^ Minority communities continue to have lower rates of ambulatory care access and quality compared with White or multiracial beneficiaries.^43^ Thus, even with the improvement of insurance coverage across the board, persistent structural and socioeconomic inequities within minority communities, some of which are suicide risk factors (eg, unemployment and lack of housing), may be limiting its effect on mortality.^30,44,45^ These factors may elucidate why our study revealed nonsignificant changes in suicide rates among Black individuals. Efforts need to be taken to mitigate the harmful mental health consequences that are on the horizon for all communities, but especially those already at a disadvantage, by investigating and addressing the persisting racial disparities in mental health, such as noted in our report.

In addition to the lack of a Medicaid expansion–associated attenuation in suicide mortality in Black individuals, there were also minimal expansion-associated changes among those aged 45 to 64 years and among women. One explanation for this variance is a difference in coverage gains between these subgroups. The key population targeted by the 2014 Medicaid expansion was nonelderly, childless, nondisabled individuals.^10^ Because disability is more prevalent in women and increases by age and because childlessness is less prevalent in women and decreases by age, women and older adults may have experienced fewer coverage gains and subsequently less drastic changes in access to mental health care and suicide rates.^46,47^ A report by Hill et al^48^ found that newly eligible individuals were more likely to be male and single compared with the pre-ACA enrollees. In addition, although the proportion of coverage-eligible individuals aged 40 to 65 years increased, the overall percentage of newly eligible individuals aged 20 to 29 years was greater than double the percentage of those aged 40 to 65 years.^48^ Thus, the lack of change in suicide rates may at least partially be attributable to lack of coverage gains among women and individuals aged 45 to 64 years.

These findings demonstrate the importance of Medicaid expansion and its benefit on a reduction in suicide in the US. Our results demonstrate an association between expansion and a mitigation in suicide mortality and supports the theory that expanding insurance coverage reduces mental health burden in the population and that Medicaid expansion prevented many unnecessary deaths by suicide.

### Limitations

The current study is limited by its ecological design. Because we do not use individual-level data, we could not assess whether the averted suicides were among new Medicaid beneficiaries or whether the completed suicides were increasingly concentrated among people who did not gain coverage through Medicaid expansion. Although we were able to account for many state-level factors, such as socioeconomic status, opioid prescribing laws, and firearm laws, factors other than Medicaid expansion may be influencing our results. For example, the state-level covariate data may inadequately account for changes at the neighborhood or individual level, and we did not have data on preexisting conditions, comorbidities, or health care use patterns for the study participants. Furthermore, the suicide data did not detail the method of suicide; thus, it is difficult to elucidate whether ACA expansion was associated with reduction in any means of suicide or whether certain means of suicide were better avoided than others. Future data will be needed to determine if the patterns observed in this study continue.

## Conclusions

Data from this cross-sectional study suggest that Medicaid expansion is associated with attenuating the increase in suicide in the US and affirm previous data that increased access to mental health care and reductions in financial barriers are vital to appropriately and adequately address the national emergency of suicides in the country. Vigorous efforts advocating to expand and improve access to mental health care should be continued.

## Notes

Supplement. **eAppendix.** Supplementary Methods **eTable 1.** MCMC Diagnostics for Hierarchical Bayesian Model **eTable 2.** Sensitivity Analyses Incorporating Group-Specific Trends **eFigure 1.** Event Study Plot to Assess Plausibility of Parallel Trends Assumption **eFigure 2.** State-Specific Temporal Trends in Age-Adjusted Suicide Rate per 100,000 for Medicaid Expansion (A) and Non-Expansion States (B)

## References

[1] National Center for Health Statistics. Suicide and self-inflicted injury. Accessed August 14, 2021. https://www.cdc.gov/nchs/fastats/suicide.htm

[2] Hedegaard H, Curtin SC, Warner M. Increase in suicide mortality in the United States, 1999-2018. NCHS Data Brief. 2020;(362):1-8. 32487287

[3] Hedegaard H, Curtin SC, Warner M. Suicide mortality in the United States, 1999-2019. NCHS Data Brief. 2021;(398):1-8. doi:10.15620/cdc:101761 33663651

[4] National Center for Injury Prevention and Control. Data and statistics (WISQARS): cost of injury reports. Accessed August 14, 2021. https://wisqars.cdc.gov/cost/

[5] US Department of Health and Human Services, Office of the Surgeon General; National Action Alliance for Suicide Prevention. *2012 National Strategy for Suicide Prevention: Goals and Objectives for Action*. US Dept of Health and Human Services; 2012. 23136686

[6] US Department of Health and Human Services, Office of the Surgeon General; National Action Alliance for Suicide Prevention. Strategic direction 3: treatment and support services. In: *2012 National Strategy for Suicide Prevention: Goals and Objectives for Action*. US Dept of Health and Human Services; 2012:50-59. 23136686

[7] US Department of Health and Human Services, Office of the Surgeon General; National Action Alliance for Suicide Prevention. Strategic direction 2: clinical and community preventive services. In: *2012 National Strategy for Suicide Prevention: Goals and Objectives for Action*. US Dept of Health and Human Services; 2012:40-49. 23136686

[8] Stone D, Holland K, Bartholow B, Crosby A, Davis S, Wilkins N. *Preventing Suicide: A Technical Package of Policy, Programs, and Practices*. Centers for Disease Control and Prevention; 2017. Accessed August 12, 2021. https://www.cdc.gov/violenceprevention/pdf/suicidetechnicalpackage.pdf

[9] Cunningham PJ. Beyond parity: primary care physicians’ perspectives on access to mental health care. Health Aff (Millwood). 2009;28(3):w490-w501. doi:10.1377/hlthaff.28.3.w490 19366722

[10] Simon K, Soni A, Cawley J. The impact of health insurance on preventive care and health behaviors: evidence from the first two years of the ACA Medicaid expansions. J Policy Anal Manage. 2017;36(2):390-417. doi:10.1002/pam.21972 28378959

[11] Saloner B, Bandara S, Bacchuber M, Barry CL. An update on “insurance coverage and treatment use under the Affordable Care Act among adults with mental and substance use disorders.” Psychiatr Serv. 2017;68(3):310-311. doi:10.1176/appi.ps.201600566 28240146

[12] Creedon TB, Cook BL. Access to mental health care increased but not for substance use, while disparities remain. Health Aff (Millwood). 2016;35(6):1017-1021. doi:10.1377/hlthaff.2016.0098 27269017 PMC7033262

[13] Winkelman TNA, Chang VW. Medicaid expansion, mental health, and access to care among childless adults with and without chronic conditions. J Gen Intern Med. 2018;33(3):376-383. doi:10.1007/s11606-017-4217-5 29181792 PMC5834959

[14] Miller S, Wherry LR. Health and access to care during the first 2 years of the ACA Medicaid expansions. N Engl J Med. 2017;376(10):947-956. doi:10.1056/NEJMsa1612890 28273021

[15] Sommers BD, Blendon RJ, Orav EJ, Epstein AM. Changes in utilization and health among low-income adults after Medicaid expansion or expanded private insurance. JAMA Intern Med. 2016;176(10):1501-1509. doi:10.1001/jamainternmed.2016.4419 27532694

[16] Sommers BD, Baicker K, Epstein AM. Mortality and access to care among adults after state Medicaid expansions. N Engl J Med. 2012;367(11):1025-1034. doi:10.1056/NEJMsa1202099 22830435

[17] Bhatt CB, Beck-Sagué CM. Medicaid expansion and infant mortality in the United States. Am J Public Health. 2018;108(4):565-567. doi:10.2105/AJPH.2017.304218 29346003 PMC5844390

[18] Eliason EL. Adoption of Medicaid expansion is associated with lower maternal mortality. Womens Health Issues. 2020;30(3):147-152. doi:10.1016/j.whi.2020.01.005 32111417

[19] Barnes JM, Johnson KJ, Boakye EA, . Early Medicaid expansion and cancer mortality. J Natl Cancer Inst. 2021;djab135. doi:10.1093/jnci/djab135 34259321 PMC8634305

[20] Khan SS, Lloyd-Jones DM, Carnethon M, Pool LR. Medicaid expansion and state-level differences in premature cardiovascular mortality by subtype, 2010-2017. Hypertension. 2020;76(5):e37-e38. doi:10.1161/HYPERTENSIONAHA.120.15968 32951471 PMC7544679

[21] Kumar SR, Khatana SAM, Goldberg D. Impact of Medicaid expansion on liver-related mortality. Clin Gastroenterol Hepatol. 2022;20(2):419-426.e1. doi:10.1016/j.cgh.2020.11.042 33278572 PMC8672394

[22] Miller S, Johnson N, Wherry LR. Medicaid and mortality: new evidence from linked survey and administrative data. Q J Econ. 2021;136(3):1783-1829. doi:10.1093/qje/qjab004

[23] Austin AE, Naumann RB, Short NA. Association between Medicaid expansion and suicide mortality among nonelderly US adults. Am J Epidemiol. 2021;190(9):1760-1769. doi:10.1093/aje/kwab130 34467410 PMC12931419

[24] Wing C, Simon K, Bello-Gomez RA. Designing difference in difference studies: best practices for public health policy research. Annu Rev Public Health. 2018;39:453-469. doi:10.1146/annurev-publhealth-040617-013507 29328877

[25] Fry CE, Hatfield LA. Birds of a feather flock together: Comparing controlled pre-post designs. Health Serv Res. 2021;56(5):942-952. doi:10.1111/1475-6773.13697 34212387 PMC8522572

[26] Case A, Deaton A. Deaths of Despair and the Future of Capitalism. Princeton University Press; 2020.

[27] Olfson M, Cosgrove C, Altekruse SF, Wall MM, Blanco C. Deaths of despair: adults at high risk for death by suicide, poisoning, or chronic liver disease in the US. Health Aff (Millwood). 2021;40(3):505-512. doi:10.1377/hlthaff.2020.01573 33646867

[28] Petterson S, Westfall JM, Miller BF. Projected deaths of despair from COVID-19. Accessed April 14, 2022. https://www.communitycommons.org/entities/36623540-a57f-40e4-955c-65aadc3e4744

[29] Huskamp HA. Mental health insurance parity: how full is the glass? In: Goldman H, Frank R, Morrissey J, eds. The Palgrave Handbook of American Mental Health Policy. Palgrove Macmillan; 2020:367-387. doi:10.1007/978-3-030-11908-9_13

[30] Robertson-Preidler J, Trachsel M, Johnson T, Biller-Andorno N. The Affordable Care Act and recent reforms: policy implications for equitable mental health care delivery. Health Care Anal. 2020;28(3):228-248. doi:10.1007/s10728-020-00391-0 32103383

[31] Stewart MT, Horgan CM, Hodgkin D, . Behavioral health coverage under the Affordable Care Act: what can we learn from marketplace products? Psychiatr Serv. 2018;69(3):315-321. doi:10.1176/appi.ps.201700098 29241429 PMC5832546

[32] Maxwell J, Bourgoin A, Lindenfeld Z. Battling the mental health crisis among the underserved through State Medicaid reforms. Accessed April 17, 2022. https://www.healthaffairs.org/do/10.1377/forefront.20200205.346125/full/

[33] Frank RG, Beronio K, Glied SA. Behavioral health parity and the Affordable Care Act. J Soc Work Disabil Rehabil. 2014;13(1-2):31-43. doi:10.1080/1536710X.2013.870512 24483783 PMC4334111

[34] Wisk LE, Peltz A, Galbraith AA. Changes in health care–related financial burden for US families with children associated with the Affordable Care Act. JAMA Pediatr. 2020;174(11):1032-1040. doi:10.1001/jamapediatrics.2020.3973 32986093 PMC7522777

[35] Griffith K, Evans L, Bor J. The Affordable Care Act reduced socioeconomic disparities in health care access. Health Aff (Millwood). 2017;36(8):1503-1510. doi:10.1377/hlthaff.2017.0083 28747321 PMC8087201

[36] Harvard TH Chan School of Public Health. Gun prevalence and suicide rank by state. Accessed November 14, 2021. https://www.hsph.harvard.edu/news/magazine/spr08gunprevalence/

[37] Guy GP Jr, Zhang K, Bohm MK, . Vital signs: changes in opioid prescribing in the United States, 2006-2015. MMWR Morb Mortal Wkly Rep. 2017;66(26):697-704. doi:10.15585/mmwr.mm6626a4 28683056 PMC5726238

[38] Barnes JM, Graboyes EM, Adjei Boakye E, . The Affordable Care Act and suicide incidence among adults with cancer. J Cancer Surviv. Published online April 4, 2022. doi:10.1007/s11764-022-01205-z 35368225

[39] Buchmueller TC, Levinson ZM, Levy HG, Wolfe BL. Effect of the Affordable Care Act on racial and ethnic disparities in health insurance coverage. Am J Public Health. 2016;106(8):1416-1421. doi:10.2105/AJPH.2016.303155 27196653 PMC4940635

[40] Baumgartner JC, Collins SR, Radley DC, Hayes SL. How the Affordable Care Act has narrowed racial and ethnic disparities in access to health care. Accessed April 14, 2022. https://www.commonwealthfund.org/publications/2020/jan/how-ACA-narrowed-racial-ethnic-disparities-access

[41] Chaudry A, Jackson A, Glied S. Did the Affordable Care Act reduce racial and ethnic disparities in health insurance coverage? Accessed April 14, 2022. https://www.commonwealthfund.org/publications/issue-briefs/2019/aug/did-ACA-reduce-racial-ethnic-disparities-coverage

[42] Mahajan S, Caraballo C, Lu Y, . Trends in differences in health status and health care access and affordability by race and ethnicity in the United States, 1999-2018. JAMA. 2021;326(7):637-648. doi:10.1001/jama.2021.9907 34402830 PMC8371573

[43] Johnston KJ, Hammond G, Meyers DJ, Joynt Maddox KE. Association of race and ethnicity and Medicare program type with ambulatory care access and quality measures. JAMA. 2021;326(7):628-636. doi:10.1001/jama.2021.10413 34402828 PMC8371568

[44] Churchwell K, Elkind MSV, Benjamin RM, ; American Heart Association. Call to action: structural racism as a fundamental driver of health disparities: a Presidential Advisory from the American Heart Association. Circulation. 2020;142(24):e454-e468. doi:10.1161/CIR.0000000000000936 33170755

[45] Bailey ZD, Krieger N, Agénor M, Graves J, Linos N, Bassett MT. Structural racism and health inequities in the USA: evidence and interventions. Lancet. 2017;389(10077):1453-1463. doi:10.1016/S0140-6736(17)30569-X 28402827

[46] Fremstad S. The Black-White disability gap increases with age. Center for Economic and Policy Research; 2020. Accessed April 21, 2022. https://cepr.net/the-black-white-disability-gap-increases-with-age/

[47] Martinez GM, Daniels K, Febo-Vazquez I. Fertility of men and women aged 15-44 in the United States: National Survey of Family Growth, 2011-2015. Natl Health Stat Report. 2018;113(113):1-17. 30248009

[48] Hill SC, Abdus S, Hudson JL, Selden TM. Adults in the income range for the Affordable Care Act’s Medicaid expansion are healthier than pre-ACA enrollees. Health Aff (Millwood). 2014;33(4):691-699. doi:10.1377/hlthaff.2013.0743 24670269

