# Supplementary material for: Association of State Medicaid Expansion Status With Rates of Suicide Among US Adults
Source: JAMA Netw Open. 2022 Jun 15;5(6):e2217228. doi: 10.1001/jamanetworkopen.2022.17228 (PMC9201676; doi:10.1001/jamanetworkopen.2022.17228)
Supplement: Supplement. — eAppendix. Supplementary Methods eTable 1. MCMC Diagnostics for Hierarchical Bayesian Model eTable 2. Sensitivity Analyses Incorporating Group-Specific Trends eFigure 1. Event Study Plot to Assess Plausibility of Parallel Trends Assumption eFigure 2. State-Specific Temporal Trends in Age-Adjusted Suicide Rate per 100,000 for Medicaid Expansion (A) and Non-Expansion States (B) [file jamanetwopen-e2217228-s001.pdf]

# Supplementary Online Content

Patel H, Barnes J, Osazuwa-Peters N, Bierut LJ. Association of state Medicaid expansion status with rates of suicide among US adults. *JAMA Netw Open*. 2022;5(6):e2217228. doi:10.1001/jamanetworkopen.2022.17228

**eAppendix.** Supplementary Methods

**eTable 1.** MCMC Diagnostics for Hierarchical Bayesian Model

**eTable 2.** Sensitivity Analyses Incorporating Group-Specific Trends

**eFigure 1.** Event Study Plot to Assess Plausibility of Parallel Trends Assumption

**eFigure 2.** State-Specific Temporal Trends in Age-Adjusted Suicide Rate per 100,000 for Medicaid Expansion (A) and Non-Expansion States (B)

This supplementary material has been provided by the authors to give readers additional information about their work.

## eAppendix 1: Supplementary Methods

*Data Format*

We utilized mortality rate data, which captures the number of suicides within a given population at a given point in time. For our analyses, suicide mortality data were obtained at the state level for all states and D.C. for each year from 2000 – 2018 and for each age group (20-29, 30-44, 45-64). This results in 19 years * 51 states (including D.C.) * 3 age groups = 2907 "observations," each of which includes the number of suicides, the number of the population, and the age-adjusted suicide rate and associated standard error for state *i*, year *t*, and age group *j*. The population included in the denominator of the rate (# suicides / # population) represents the entire U.S. population ages 20-64 years, and the suicide mortality data encompass all reported suicides (based on ICD codes on death certificates) within this population. Due to the nature of this data format, conceptualizing the concept of "sample size" is difficult. One could view all individuals ages 20-64 years living in the U.S. from 2000 – 2018 as the sample given their inclusion in the suicide rate calculations. Alternatively, since data are reported at the state level, one could also view the sample size as 51, one for each state, with repeated measures over time. We prefer to report sample size in terms of the number of unique observational units (at the state-age-year level), which in our case is 2907.

*Covariates*

As above, data were obtained by state, year, and age group. These factors were all included in our model as covariates. Year was treated as a linear continuous variable (rescaled such that the year 2014 was 0). Due to concerns of heterogeneous trends in suicide rates over time by state, we also included a state*year interaction effects. Further information regarding the selection of the state-level trends is presented in the statistical analysis section.

© 2022 Patel H et al. *JAMA Network Open.*

We obtained time-varying (including both pre-and post-expansion values) state-level covariates, which were selected a priori due to suspected associations with suicide mortality and/or access to care. Census estimates from the United States Census Bureau were used to define race/ethnicity (% non-Hispanic white) data in 2010 (pre-expansion) and 2020 (post-expansion).[1,2] State-level poverty (% of individuals living in poverty based on the federal poverty level definition) data in 2000, 2010, 2015, and 2019 were obtained using US Census Bureau Small Area Income and Poverty Estimates.[3] U.S. Department of Agriculture Economic Research Service (USDA ERS) databases were utilized to obtain state-level education (% without at least a high school education) based on 2000 and the 2013-17 average data as well as yearly unemployment (% unemployed) from 2007-2017.[4] State-level data was then merged with the mortality data by state and year (or nearest matching year). The number of state opioid prescribing laws and firearm laws were obtained from recent publications.[5–7]

*Difference-in-Differences Analyses*

The unadjusted DID estimate is defined as

$$\text{DID} = (\text{Rate}_{\text{Expansion State,Post-ACA}} - \text{Rate}_{\text{Expansion State,Pre-ACA}})$$
$$- (\text{Rate}_{\text{Non-expansion State,Post-ACA}} - \text{Rate}_{\text{Non-expansion,Pre-ACA}}).$$

It can be shown that the DID estimate is equivalent to the interaction effect between state Medicaid expansion status (expansion vs. non-expansion) and time period (pre- vs. post-expansion) in a linear regression model, which also enables adjustment for other factors and modeling of correlation between observations.[8] We use a flexible hierarchical Bayesian regression framework for our linear regression approach to difference-in-differences to overcome some of the challenges associated with these data. Our modeling approach is largely based on a

© 2022 Patel H et al. *JAMA Network Open.*

previously reported method.[9] For the sake of completeness of our methodology, we include that information here:

With observed vector of mortality rates $\boldsymbol{Y_i}$ and vector of associated estimated variances $\boldsymbol{s_i^2}$ for state *i*, age group *j*, and year group *t*, diagonal matrix $S_i$ containing the standard deviations $\boldsymbol{s_i}$, correlation matrix R, mortality rate average for the national population of interest *m*, and covariates *k*=1, …, K, we developed a hierarchical model such that:

$\boldsymbol{Y_i} \sim MVN(\underline{\mu} + \mathbf{X_i} * \boldsymbol{\beta}, \Sigma_K)$, $\Sigma_K = S_i R S_i$ , R = correlation matrix with off-diagonal elements = ρ

$\mu \sim N(m, a=100)$

$\boldsymbol{\beta} \sim N(\mathbf{0}, diag(\sigma_1^2, \ldots, \sigma_K^2))$

$\sigma_k \sim Cauchy(0, 2.5)\ T(0, Infinity)$

$\rho \sim Uniform(l,u)$

Suicide mortality rates were assumed to follow a normal distribution by the central limit theorem, as the rate can be viewed as a mean.[10] However, the variance for the mortality rates were not be equal across state-age-year units given the differences in population and number of observed events. A prior was not placed for these values given that the estimated variance is simply a function of other values. We utilize a multivariate normal distribution to obtain all draws for a given state (across all years *t* and age groups *j*) to be able to explicitly model the correlation from the repeated measures from the state. The correlation matrix R was a 57 * 57 (note 57 = 19 years * 3 age groups) symmetric matrix with diagonal elements equal to 1 and off-diagonal elements equal to ρ. Note that such a structure is considered an exchangeable correlation matrix. Such a specification was based on empiric estimates of the correlation matrix, which showed remarkably similar correlations across years that did not diminish over time or

© 2022 Patel H et al. *JAMA Network Open.*

across age groups. The values l and u for the flat prior for ρ were also based on the empiric estimates of the correlation matrix and were determined to 0.4 and 0.9, respectively, corresponding to the range of values within the estimated correlation matrix. Note that specifying an independent variance matrix (i.e. no correlation component) gave in very similar results (data not shown), perhaps due to the use of the year*state interaction terms to allow for state-specific temporal trends (which, in the Bayesian framework where all parameters are treated as random variables, function similarly to random intercepts and random slopes in mixed effects models, though without the distinction between fixed and random effects). Note that the use of the multivariate normal distribution provides two distinct advantages over potential binomial approaches: (1) the use of linear regression, which is often preferable in DID analyses,[11] and (2) the ability to directly incorporate correlation structures accounting for autocorrelation into the likelihood for the response variable.

The prior for μ was selected to be centered roughly around the corresponding suicide mortality rate for the nation over the study period, however with much greater variance. We specified m=16 for the overall and age subgroup analyses, and used m=19, 10, 12, 28, and 8 for subgroup analyses for White, Black, Other, males, and females, respectively. The variance for the prior for μ was set to *a*=100 (corresponding to standard deviation of 10) for all analyses. The prior for **β** is typical for regression analyses. The prior for $\sigma_k$ was a half Cauchy distribution, as recommended by Gelman et al.,[12] given its precision with standard deviations closer to 0 and its ability to accommodate much larger standard deviations should the need arise. Note that the prior distribution for the $\beta_k$ (assuming the null, as specified incorporating the $\sigma_k$) has ~75% of its density between -5 and 5, over 10% of its density with values more extreme than 10 (or -10), and

© 2022 Patel H et al. *JAMA Network Open.*

still over 1% of its density for values more extreme than 100 (or -100), which was deemed appropriate for the effect sizes expected with the present data.

Draws from the posterior distribution were obtained with Gibbs sampling via R2jags. We obtained 20,000 draws and discarded the first 2,000 as burn in. Convergence of the draws were assessed visually and with the Geweke, Raftery and Lewis, and Heidelberger and Welch diagnostics. Additional draws were discarded as burn in as necessary (note eTable 1 footnotes) based on chain diagnostic criteria. A summary of those tests for the posterior distribution of the DID estimator (the interaction between time period and Medicaid expansion status) are given in eTable 1.

References

1. United States Census Bureau. County Intercensal Datasets: 2000-2010. https://www.census.gov/data/datasets/time-series/demo/popest/intercensal-2000-2010-counties.html. Accessed September 14, 2020.
2. United States Census Bureau. County Population by Characteristics: 2010-2018. https://www.census.gov/data/tables/time-series/demo/popest/2010s-counties-detail.html#. Published 2020. Accessed May 7, 2020.
3. United States Census Bureau. Small Area Income and Poverty Estimates (SAIPE) Program. https://www.census.gov/programs-surveys/saipe/data/datasets.html. Accessed September 14, 2020.
4. US Department of Agriculture Economic Research Service. USDA ERS - County-level Data Sets. https://www.ers.usda.gov/data-products/county-level-data-sets/. Accessed January 3, 2020.

© 2022 Patel H et al. *JAMA Network Open.*

5. Meara E, Horwitz JR, Powell W, et al. State Legal Restrictions and Prescription-Opioid Use among Disabled Adults. *N Engl J Med*. 2016;375(1):44-53. doi:10.1056/NEJMsa1514387

6. Bulls HW, Bell LF, Orris SR, et al. Exemptions to state laws regulating opioid prescribing for patients with cancer-related pain: A summary. *Cancer*. 2021;127(17):3137-3144. doi:10.1002/cncr.33639

7. Siegel M, Pahn M, Xuan Z, et al. Firearm-related laws in all 50 US States, 1991-2016. *Am J Public Health*. 2017;107(7):1122-1129. doi:10.2105/AJPH.2017.303701

8. Dimick JB, Ryan AM. Methods for evaluating changes in health care policy: the difference-in-differences approach. *JAMA*. 2014;312(22):2401-2402. doi:10.1001/jama.2014

9. Barnes JM, Johnson KJ, Adjei Boakye E, et al. Early Medicaid Expansion and Cancer Mortality. *JNCI J Natl Cancer Inst*. July 2021. doi:10.1093/jnci/djab135

10. Quick H, Waller LA, Casper M. A multivariate space–time model for analysing county level heart disease death rates by race and sex. *J R Stat Soc Ser C (Applied Stat*. 2018;67(1):291-304. doi:10.1111/rssc.12215

11. Ai C, Norton EC. Interaction terms in logit and probit models. *Econ Lett*. 2003;80(1):123-129. doi:10.1016/S0165-1765(03)00032-6

12. Gelman A. Prior distributions for variance parameters in hierarchical models (comment on article by Browne and Draper). *Bayesian Anal*. 2006;1(3):515-534. doi:10.1214/06-BA117A

© 2022 Patel H et al. *JAMA Network Open.*

**eTable 1: MCMC diagnostics for hierarchical Bayesian model**

| | Geweke Diagnostic | Heidelberger-Welch Diagnostic | | Raftery-Lewis Diagnostic |
|---|---|---|---|---|
| | Z score | Stationary Start, p | Halfwidth Test, mean (halfwidth) | $N_{chain}$ (Dependence Factor) |
| Overall | -1.069 | 0.96 | -0.40 (0.003) | 3918 |
| Female | -1.023 | 0.94 | -0.17 (0.004) | 3901 |
| Male[a] | 0.714 | 0.13 | -0.41 (0.008) | 16740 |
| 20-29 yrs[a] | -1.35 | 0.053 | -0.51 (0.009) | 4041 |
| 30-44 yrs | 0.93 | 0.56 | -0.30 (0.007) | 3901 |
| 54-64 yrs | 1.88 | 0.28 | -0.04 (0.003) | 3848 |
| White | -0.40 | 0.45 | -0.39 (0.004) | 3778 |
| Black[a] | -0.60 | 0.58 | 0.17 (0.004) | 3726 |
| Other | -1.77 | 0.69 | -0.35 (0.015) | 4216 |

[a]The chains from these analyses required additional burn-in of 1,000 draws, and age 20-29 years required removal of an additional 4,000 draws.

© 2022 Patel H et al. *JAMA Network Open.*

**eTable 2: Sensitivity analyses incorporating group-specific trends**

| | DID estimate[a] | | Group trend differential[b] | |
|---|---|---|---|---|
| | Est (95% CrI) | Pr(Est>0) | Est (95% CrI) | Pr(Est>0) |
| Overall | -0.41 (-0.67, -0.15) | 0.001 | -0.01 (-0.12, 0.09) | 0.434 |
| Female | -0.18 (-0.41, 0.05) | 0.067 | 0.01 (-0.08, 0.10) | 0.57 |
| Male | -0.39 (-0.82, 0.02) | 0.033 | -0.05 (-0.19, 0.09) | 0.26 |
| 20-29 yrs | -0.54 (-1.02, -0.05) | 0.013 | 0.04 (-0.09, 0.19) | 0.72 |
| 30-44 yrs | -0.27 (-0.70, 0.11) | 0.082 | -0.04 (-0.18, 0.09) | 0.27 |
| 45-64 yrs | -0.03 (-0.41, 0.34) | 0.44 | -0.01 (-0.15, 0.13) | 0.47 |
| White | -0.37 (-0.67, -0.06) | 0.01 | -0.13 (-0.24, -0.02) | 0.015 |
| Black | 0.15 (-0.18, 0.51) | 0.79 | 0.07 (-0.06, 0.21) | 0.85 |
| Other | -0.39 (-1.12, 0.21) | 0.13 | 0.08 (-0.13, 0.29) | 0.77 |

[a]The DID estimate, just as in other analyses, represents the interaction between state expansion status and time period.
[b]The group trend differential is equal to the coefficient for the Year * state expansion status interaction term.
CrI = Credible Interval. Note that the Pr(Est>0) is a 1-tailed probability, so Pr < 0.025 is required for statistical significance.

© 2022 Patel H et al. *JAMA Network Open.*

**eFigure 1**: Event Study Plot to Assess Plausibility of Parallel Trends Assumption

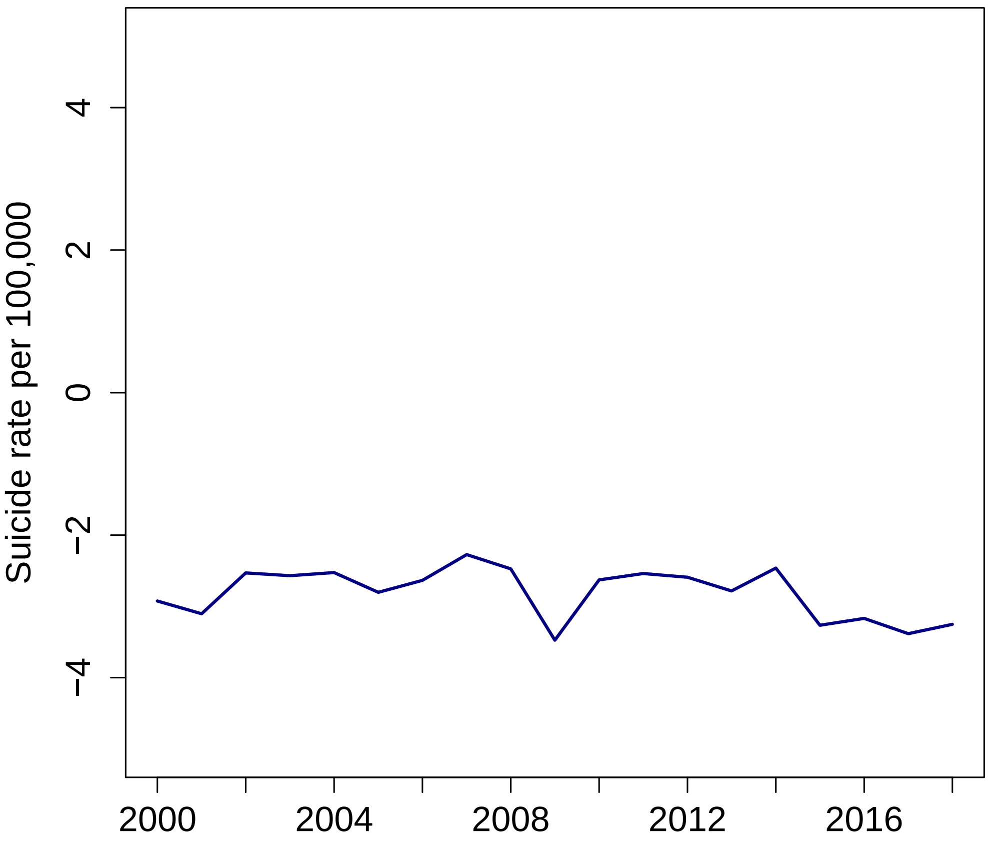


Legend: The difference in age-adjusted suicide rates between Medicaid expansion and non-expansion states is shown above. Note that the difference is relatively stable over the pre-expansion period, 2000-2014, with the exception of 2009. Note that the data utilized to generate this figure exclude states that expanded Medicaid in 2015-2018, though such states were able to be included in the primary DID analyses through the incorporation of a staggered time period effect.

© 2022 Patel H et al. *JAMA Network Open.*

**eFigure 2**: State-specific temporal trends in age-adjusted suicide rate per 100,000 for Medicaid expansion (A) and non-expansion states (B).

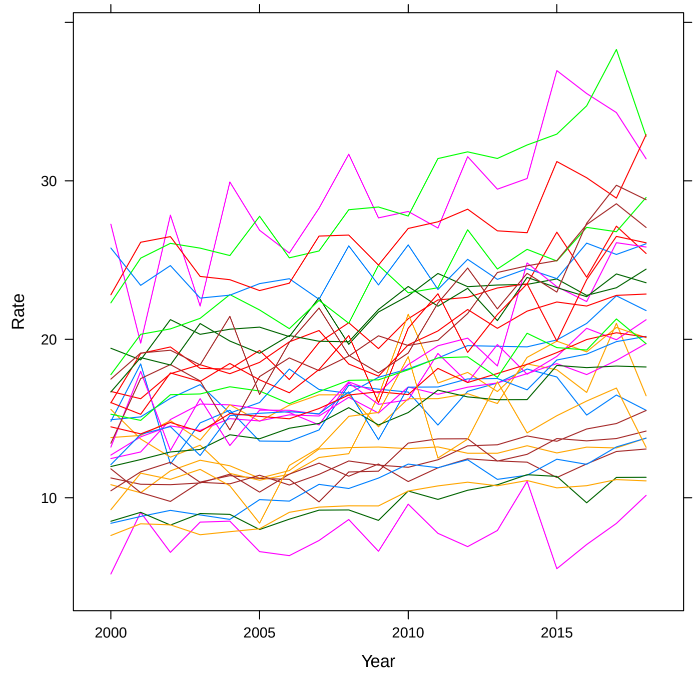


(A) Medicaid Expansion States

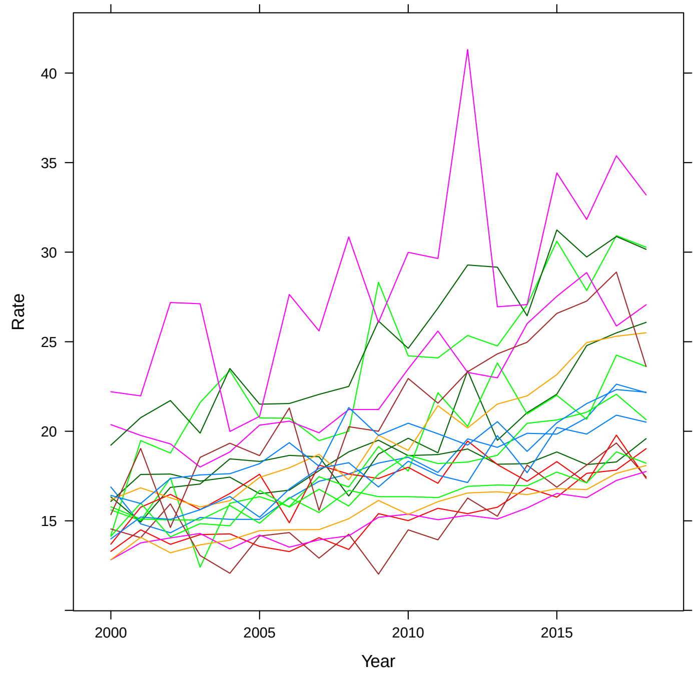


(B) Non-expansion States

Legend: Note the differing baseline levels and trajectories/slopes of the suicide rates by state.

© 2022 Patel H et al. *JAMA Network Open.*
